# Effects of HIV-1-induced CD1c and CD1d modulation and endogenous lipid presentation on CD1c-restricted T-cell activation

**DOI:** 10.1186/1471-2172-14-4

**Published:** 2013-01-24

**Authors:** Halonna Kelly, Rajakumar Mandraju, Jordana GA Coelho-dos-Reis, Moriya Tsuji

**Affiliations:** 1 HIV and Malaria Vaccine Program, Aaron Diamond AIDS Research Center, Affiliate of The Rockefeller University, 455 First Avenue, New York, NY, 10016, USA; 2 Department of Medical Parasitology, New York University School of Medicine, 341 East 25th Street, New York, NY, 10010, USA; 3 Current Address: Basic Immunology Branch, Division of Allergy, Immunology, and Transplantation, National Institute of Allergy and Infectious Diseases, National Institutes of Health, Bethesda, MD, 20892, USA

**Keywords:** CD1c, Phosphatidylcholine, Cholesterol, T cell, HIV-1, Vpu

## Abstract

**Background:**

It has been shown that human immunodeficiency virus (HIV)-1 infection induces the production of endogenous lipids required for effective viral production, and the cluster of differentiation (CD)1 molecule CD1d is downregulated by HIV-1 infection. However, the role of endogenous lipid presentation and the implications of CD1 downregulation by HIV-1 infection have not yet been characterized.

**Results:**

In this study, we observed downregulation of both CD1c and CD1d expression through a Vpu-dependent and Nef-independent mechanism, and the concomitant HIV-1-induced production of host cholesterol decreased the extent of CD1c and CD1d modulation. While the modest downregulation of CD1c by HIV-1 infection decreased the ability of CD1c-restricted T cells to respond and secrete interferon-γ, the cholesterol upregulation in the same cells by HIV-1 infection appears to limit the downregulation of CD1c.

**Conclusions:**

The two conflicting HIV-1-mediated changes in CD1c expression appear to minimize the modulation of CD1c expression, thus leading the host to maintain a CD1c-restricted T-cell response against HIV-1.

## Background

Human immunodeficiency virus (HIV)-1, the etiological agent of acquired immune deficiency syndrome (AIDS), infects nearly 40 million people worldwide. Despite numerous advances in the understanding of HIV/AIDS, attempts at inducing a major histocompatibility complex (MHC)-restricted immune response, although promising in some respects, have failed to generate a sufficient response to clear the infection. This could be due to the breadth of the response, the type of response, such as responses mediated by cytotoxic T cells versus those mediated by antibodies, or the timing of the response, such as a response that is too late to keep up with the high viral replication rate.

Cluster of differentiation (CD)1 molecules are non-polymorphic MHC-like molecules that associate with β2-microglobulin on the surface of various types of cells, particularly antigen-presenting cells (APCs). However, unlike MHC molecules that present peptide antigen to T cells, CD1 molecules have hydrophobic binding pockets that bind hydrocarbon chains making them suitable for presentation of lipid antigens [1-3]. In humans, there are five types of CD1s: CD1 a, b, and c are classified in group 1, CD1d in group 2 and CD1e in group 3. Group 1 CD1s and CD1d are recognized by CD1-restricted T cells and invariant natural killer T (*i*NKT) cells, respectively.

CD1 molecules present both endogenous and pathogen-derived lipids. For presentation of endogenous lipids, it is thought that pathogen engagement of cellular proteins, such as toll-like receptors, can induce the production of endogenous lipids, and the subsequent increase in lipid presentation by CD1 molecules induces the activation of CD1-restricted T cells [2]. Several groups have investigated the reactivity of CD1-restricted cells against both endogenous- [4-10] and pathogen- [1,7,11-15] derived lipids. Murine CD1d-restricted T-cell hybridomas recognize CD1d molecules presenting phosphatidylinositol, phosphatidylethanolamine, and phosphatidylglycerol [6]. In humans, GM1 gangliosides found in neural tissues and GD3 gangliosides found in melanomas have been shown to be recognized by CD1d-restricted T cells [1]. Sulfatide, a lipid found primarily in the brain, is thought to be a promiscuous lipid and is recognized by CD1a, b, and c- restricted T cells in humans [16]. Recently, it has been shown that CD1-restricted T cells reactive against self lipids are bi-functional, as they can also recognize foreign mycobacterial-derived lipids. These cells were shown to be CD8^+^, have an αβ T-cell receptor (TCR), and possess cytolytic activity [7]. The recognition of lipids by their CD1-restricted T cells can mediate cytokine production [4], induce maturation of dendritic cells (DCs) [17,18], and activate other cells in the immune response [12,19].

Unlike proteins, lipids are relatively conserved. Thus, the limited variability of lipids suggests that CD1-restricted T cells could play an important role in the immune response long before MHC-restricted T cells and B cells have had a chance to expand. In line with this, studies that assessed the effects of viral infection on CD1 expression showed that CD1d was downregulated during herpes infection [20,21], and the lack of CD1d or Jα18 *i*NKT cells resulted in an impaired capacity to clear the infection [22]. HIV-1 has also been shown to downregulate CD1d expression and antigen-presentation capabilities [23-27]. A few studies indicate that this phenomenon is caused in part through interactions between the Nef protein and the cytoplasmic tail of the CD1d molecule in a manner similar to the downregulation of MHC-class I and CD4 molecules [23,24]. However, one study showed that Nef did not bind to or alter the expression of molecules with the CD1d cytoplasmic tail [26]. Another recent study showed the inhibition of CD1d recycling from endosomal compartments to the cell surface by interactions between Vpu and CD1d molecules [27]. Together, these studies suggest that the viruses downregulate the CD1 molecules in an effort to evade or delay the immune response against the infection. However, to date, the consequences of this downregulation have not been studied in greater detail.

Lipid rafts are specialized membrane domains enriched in certain lipids, cholesterol, and proteins. The HIV-1 gag protein associates with lipid rafts to facilitate viral budding. Cholesterol plays a central role in maintaining the function of these rafts, and the depletion of cholesterol markedly reduces HIV-1 virion production [28]. Microarray analysis of infected cells has revealed that the presence of HIV-1 increases the expression of cholesterol genes and the incorporation of cholesterol precursors, and these effects are mediated by Nef [29,30]. These studies suggest that endogenous lipids may play a vital role in sustaining the viral life cycle within the host and/or enhancing viral productivity. However, it is not yet known whether the increase in endogenous lipid production induced by HIV-1 infection can also result in an increase in the presentation of these lipids by CD1 on the cell surface to signal the immune system that an infection has occurred. We sought to address the role of endogenous lipid presentation during HIV-1 infection. To accomplish this, we assessed how increased cholesterol production affects CD1c expression and the activation of CD1c-restricted T cells, how the modulation of CD1c expression is triggered by HIV-1 infection, and how modulation of CD1c expression by HIV-1 affects CD1c-restricted T-cell activation and consequently viral production.

## Results

### Characterization of CD1c-restricted T cells

**Figure 1:**
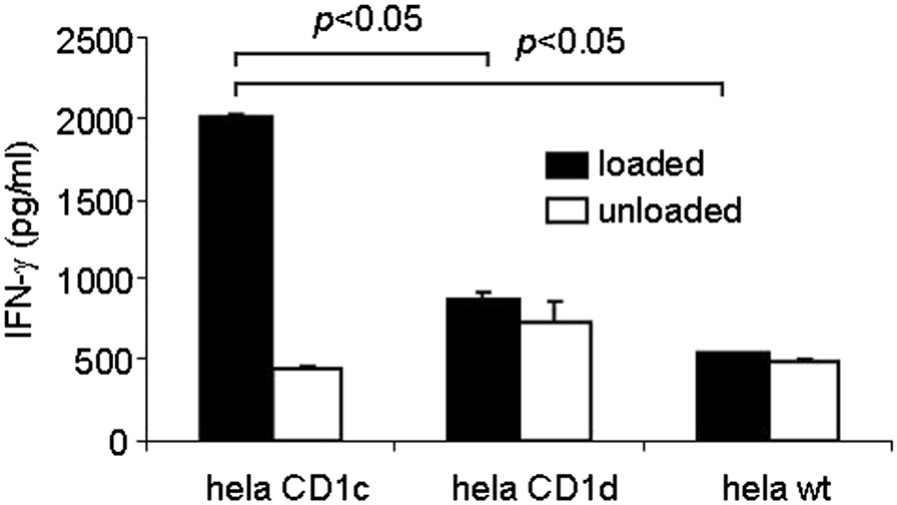
**CD1c-restricted T cells are reactive against the endogenous lipid phosphatidylcholine.** HeLa cells were transfected with CD1c and CD1d constructs and used as antigen presenting cells. The HeLa-CD1 cells were loaded with 50 μg/ml phosphatidylcholine (PC) and used to stimulate CD1c-restricted T cells for 24 hours. Supernatants were collected and used in an IFN-γ ELISA. Data represent results from one of three independent experiments.

To generate CD1c-restricted T cells, human T cells were isolated from healthy donors and stimulated for several weeks with autologous DCs loaded with the endogenous lipid phosphatidylcholine (PC). These cells were then tested for functionality, CD1c-restriction, and phenotyped. The analysis indicated that these CD8^+^, αβ TCR, non-cytotoxic (data not shown) T cells are PC-reactive and CD1c-restricted (Figure 1).

### Expression levels of CD1c/CD1d molecules during increased lipid synthesis

**Figure 2:**
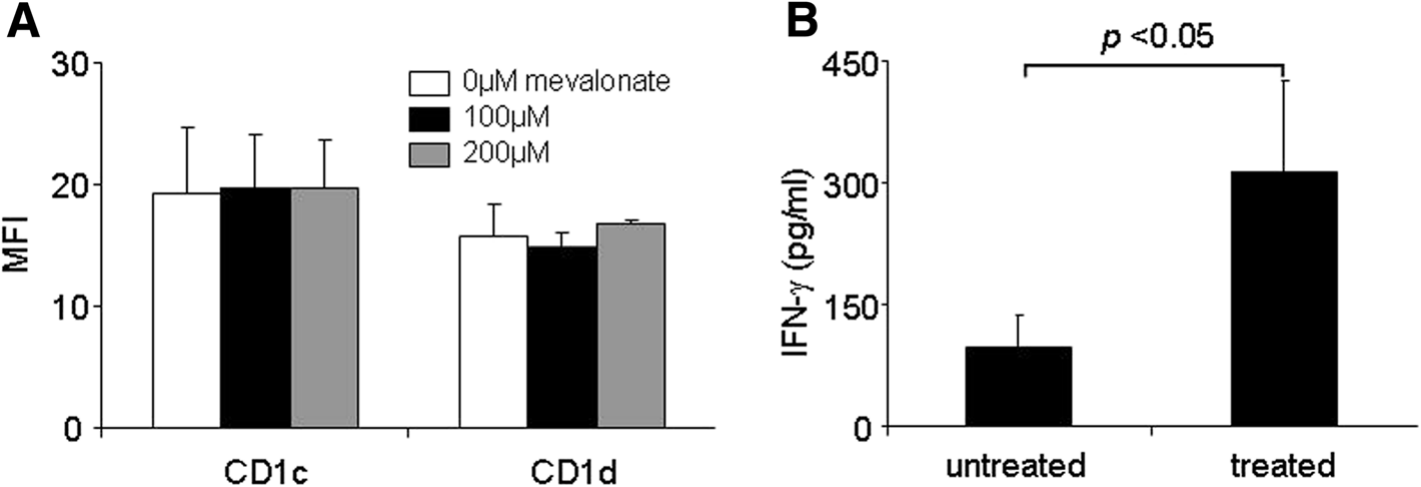
**Increased cholesterol production does not alter CD1c/CD1d expression, but induces IFN-γ in CD1c-restricted T cells.** (**A**) Jurkat cells were cultured in the presence of varying amounts of mevalonate, a precursor and inducer of cholesterol. CD1c and CD1d expression was assessed 10 days later via FACS. Data were averaged from three independent experiments. (**B**) Jurkat cells were either untreated or cultured with 200 μM mevalonate for 10 days and then used to stimulate CD1c-restricted T cells for 24 hours. Supernatants were collected and analyzed by IFN-γ ELISA. Standard deviation bars are averaged from three separate experiments.

HIV-1 infection induces the production of cholesterol [29,30], which may be presented by CD1c/CD1d molecules to initiate an immune response. To address this issue, we determined whether increased lipid production results in increased CD1c/CD1d expression. To accomplish this, Jurkat cells were cultured for 10 days in the presence of mevalonate, a precursor and known cholesterol inducer. The measurement of cholesterol production revealed that the treated cells produced significantly more cholesterol than untreated controls (Table 1). However, despite increased cholesterol production, the CD1c/CD1d levels remained unchanged (Figure 2A), suggesting that increased lipid synthesis does not result in increased CD1c/CD1d expression.

**Table 1:** Cholesterol measurement in treated and HIV infected cells

**Total cholesterol (μg/ml)**		
	**Average**	**S.D.**
(−)	21.26	4.10
treated	93.51*	5.57
infected	50.28^#^	13.63

* p < 0.05, ^#^ p < 0.05.

Total cell cholesterol was measured in Jurkat cells *untreated* or *treated* with 200 μM mevalonate or *infected* with HIV IIIB using the Amplex Red Cholesterol Kit, as described. * Indicates a significant difference between the untreated and the mevalonate treated cells. ^#^ Indicates a significant difference between untreated cells and HIV infected cells. Data represent results from one of three independent experiments.

### Effects of increased cholesterol on CD1c-restricted T-cell responses

We next wanted to know whether cells producing excess cholesterol were more capable of inducing CD1c-restricted T cells to secrete interferon (IFN)-γ compared to cells not producing excess lipid. Jurkat cells were treated with 200 μM of mevalonate and used to stimulate the secretion of IFN-γ from CD1c-restricted T cells. The cells treated with mevalonate had an enhanced capacity to stimulate CD1c-restricted T cells compared to untreated controls (Figure 2B), suggesting that CD1c-restricted T cells may be able to recognize increased lipid levels in the context of CD1c molecules.

### Effects of HIV-1 infection on CD1c/CD1d expression

**Figure 3:**
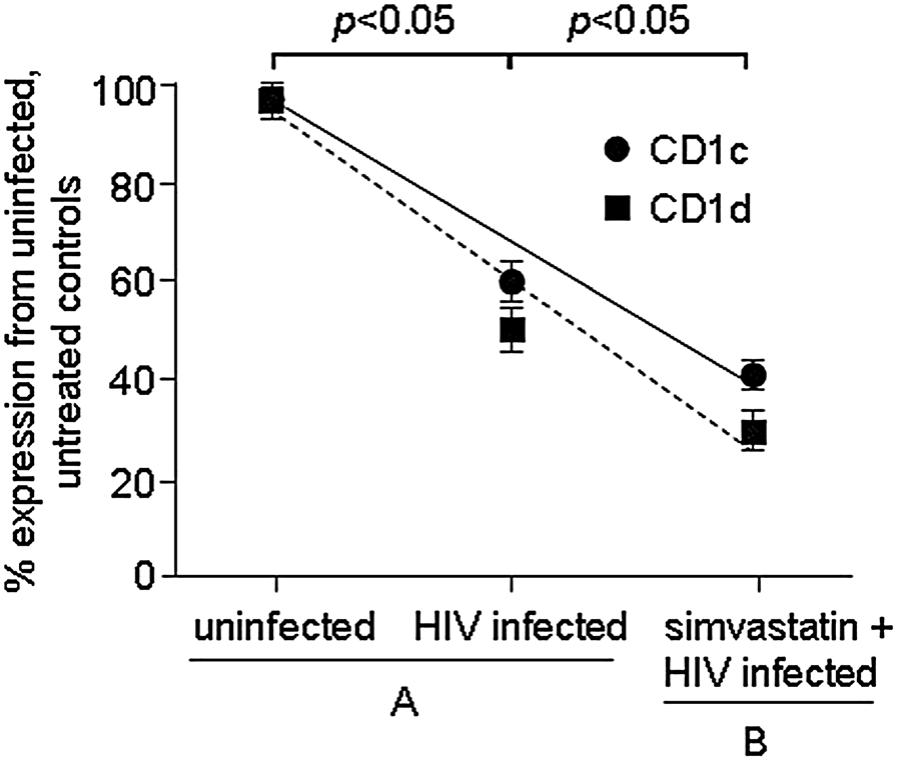
**Effects of HIV-1 on CD1c/CD1d expression.(A**) HIV-1 infection modulates CD1c/CD1d expression. Jurkat cells were mock or HIV-1 IIIb infected for 10 days, and the levels of CD1c and CD1d expression were measured by FACS. CD1c/CD1d expression was normalized using uninfected, treated controls and plotted as % modulation from the controls. (**B**) Blocking cholesterol production results in significant CD1c/CD1d downregulation during HIV-1 infection. Jurkat cells were either treated with simvastatin, an inhibitor of cholesterol synthesis, or infected with HIV IIIb and cultured for 5 days. The levels of CD1c and CD1d expression were assessed by FACS, and data were normalized using uninfected, treated controls and plotted as % modulation from controls. All data represent results from one of three independent experiments.

Based on our findings that an increase in cholesterol production resulted in increased stimulation of CD1c-restricted T cells and studies by other groups showing that HIV induces cholesterol production [28,29], we next assessed the level of CD1c expression during HIV-1 infection. For this purpose, we infected Jurkat cells with HIV-1 IIIb and measured CD1c/CD1d expression by FACS on day 10. We found that CD1c was downregulated 40% compared to uninfected controls, while CD1d was downregulated by 53% on average from the cell surface (Figure 3A). Similar levels of CD1c/CD1d modulation were found with varying viral titrations, suggesting that the viral titer does not significantly affect the levels of CD1c and CD1d downregulation (data not shown).

### Effects of Nef and Vpu on CD1c/CD1d expression

**Figure 4:**
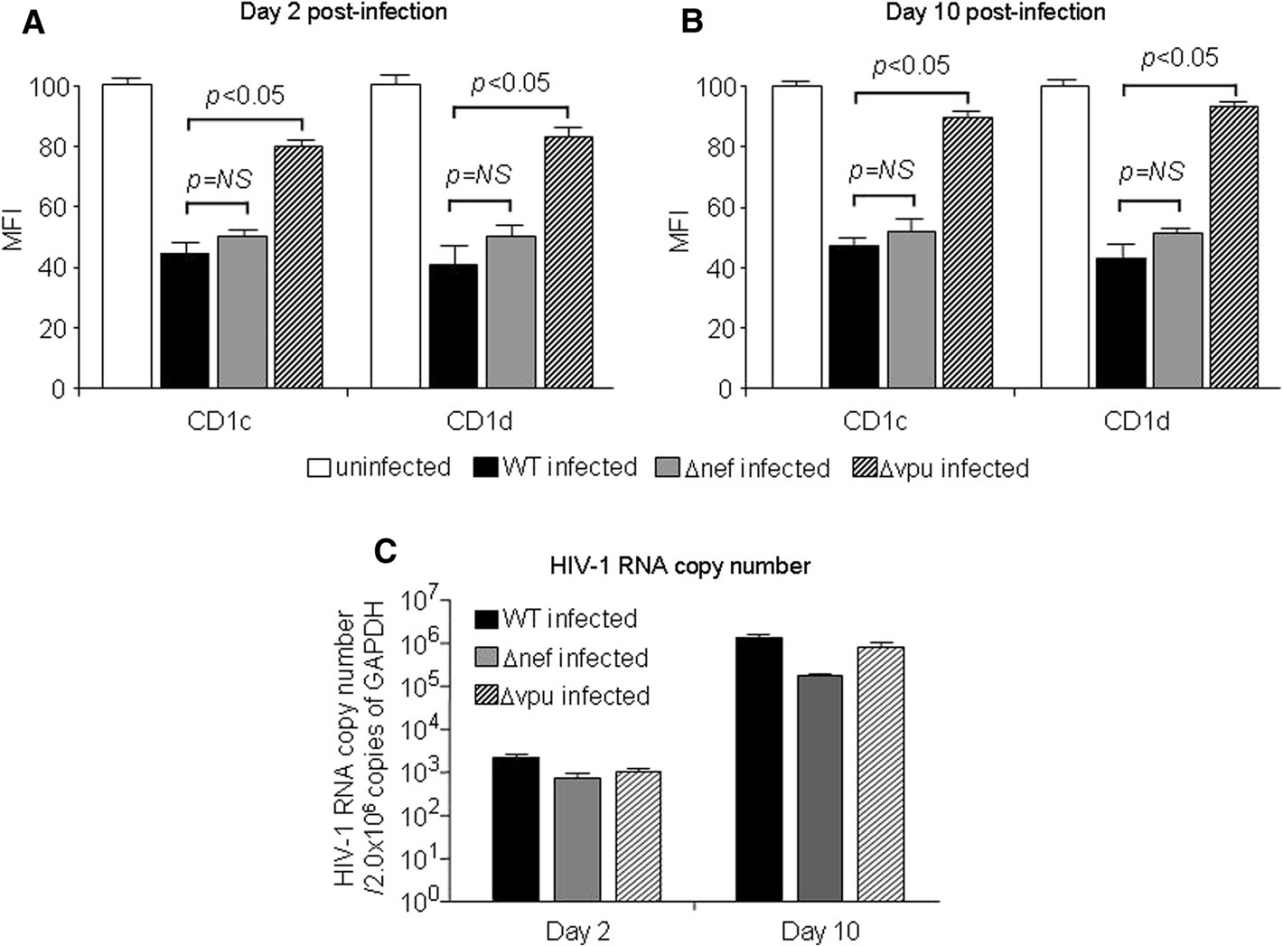
**Vpu, but not Nef, plays a role in CD1c/CD1d modulation.** Jurkat cells were infected with wild-type (WT), delta Vpu (ΔVpu), or delta Nef (ΔNef) NL4-3 viruses for 2 days (**A**) or 10 days (**B**). The expression levels of CD1c and CD1d were then determioned by FACS and expressed as Mean Fluorescence Intensity (MFI). Data represent results from one of three independent experiments. (**C**) HIV-1 RNA copy number at each time point (2 days and 10 days post-infection) was determined by qPCR. HIV-1 RNA copy number was normalized based on the copy number of GAPDH. Data represent results from one of three independent experiments.

The mechanism of CD1 molecule modulation during HIV-1 infection is poorly understood. While some have shown that CD1d is downregulated by Nef [23,24], one study showed that Nef does not alter the expression of CD1d [26], and another showed that CD1d expression is downregulated by Vpu [27]. When we infected Jurkat cells with wild-type (WT), Nef-deleted (ΔNef) or Vpu-deleted (ΔVpu) viruses, we found a significant difference between the WT and ΔVpu-infected cells, but not the WT and ΔNef infected cells on days 2 (Figure 4A) and 10 (Figure 4B) post-infection. The viral RNA copy numbers among different viruses were similar (Figure 4C). In fact, the viral load of ΔVpu virus appeared to be slightly higher than that of ΔNef virus (Figure 4C), ensuring that the CD1 down-modulation was not due to the amounts of viruses used to infect Jurkat cells.

**Figure 5:**
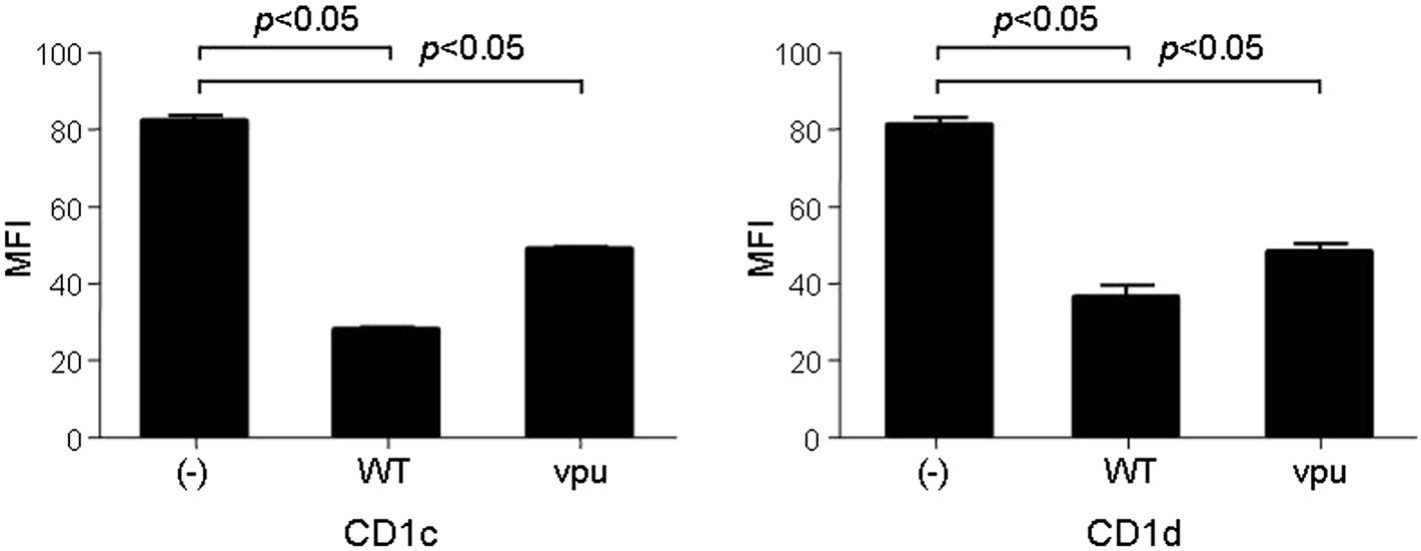
**Vpu-dependent CD1c and CD1d down-modulation.** Jurkat cells were transfected with full-length HIV (WT) or Vpu-only (vpu) expressing vectors and CD1c and CD1d expression was checked after 24 hours of transfection. Results correspond to the level of CD1c and CD1d expression (MFI) of untransfected and transfected cells as determined by FACS.

In order to confirm whether Vpu itself is sufficient to accomplish this function, we transfected Jurkat cells with a Vpu-expressing plasmid and determined the expression of CD1c/CD1d molecules by the transfected Jurkat cells. As shown in Figure 5, the transfection of a plasmid encoding only Vpu could significantly downregulated both CD1c and CD1d albeit to a lesser degree than that caused by WT plasmid encoding a whole HIV-1 sequence.

These results altogether indicate that the levels of CD1c and CD1d expression were downregulated in a Vpu-dependent and Nef-independent fashion.

### The effect of HIV-1-induced cholesterol synthesis on CD1 expression

The measurement of cholesterol production in the HIV-1-infected cells showed a significant 2-fold increase in cholesterol production compared to uninfected controls (Table 1). Thus, these data suggest that, during HIV-1 infection, CD1c is downregulated and cholesterol production is increased. To rationalize why CD1c/CD1d modulation is not more significantly enhanced during HIV-1 infection and understand the role cholesterol production may play in CD1c/CD1d expression during infection, we blocked cholesterol production in the HIV-1-infected cells by incubating the cells with 10 μM simvastatin, an enzyme that inhibits the cholesterol biosynthesis pathway. We measured a dramatic decrease in CD1c and CD1d expression in the presence of the inhibitor (Figure 3B), with CD1c expression decreasing by 17% compared to the HIV infected, untreated controls. Simvastatin treatment alone had minimal (less than 1% modulation) effects on CD1c expression (data not shown). These data suggest that cholesterol production during HIV-1 infection may aid in preventing further decreases in CD1c and CD1d expression.

### The effect of CD1c modulation and increased lipid synthesis by HIV-1 infection on the CD1c-restricted T-cell response

**Figure 6:**
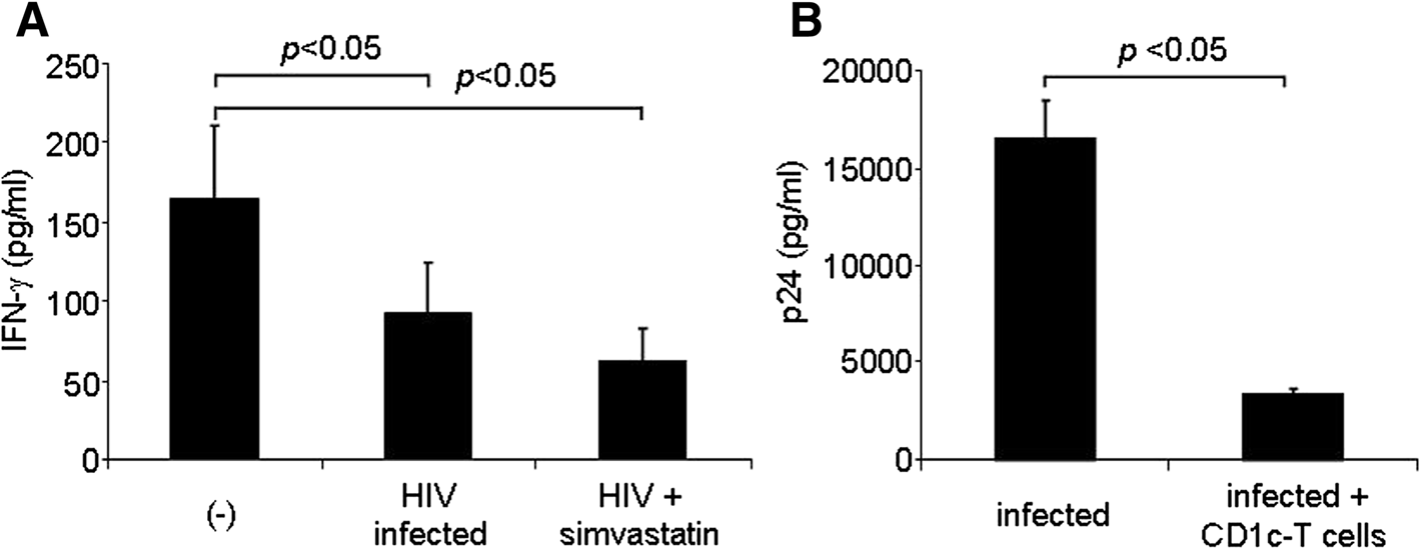
**HIV-1 infected cells have decreased capacity to induce IFN-γ secretion by CD1c-restricted T cells.** (**A**) Jurkat cells were either mock or HIV-1 IIIb infected with or without 10 μM simvastatin for 10 days. The cells were then used to stimulate CD1c-restricted T cells for 24 hours. Supernatants were collected and subjected to IFN-γ ELISA. (**B**) HIV-infected cells were cultured with or without CD1c-restricted T cells overnight, and p24 was subsequently measured in supernatants. These data represent results from one of three independent experiments.

Because an increased production of cholesterol resulted in increased stimulation of CD1c-restricted T cells, and HIV-1 infection induced the downregulation of CD1c expression, we hypothesized that the HIV-1-mediated downregulation of CD1c and elevated cholesterol production would counteract each other and lead to the overall level of the CD1c-restricted T-cell response. To test this, we cultured HIV-1-infected Jurkat cells with CD1c-restricted T cells and measured IFN-γ production by the CD1c-restricted T cells. Compared to uninfected controls, the HIV-1 infected cells showed a decreased capacity to activate CD1c-restricted T cells (Figure 6A). Thus, the decrease in CD1c expression during HIV-1 infection appears to affect the ability of CD1c-restricted T cells to recognize infected cells. This decrease in the CD1c-restricted T-cell response was even more pronounced in the presence of the cholesterol inhibitor simvastatin, where CD1c expression was further decreased.

### Anti-HIV-1 activity by CD1c-restricted T cells during HIV-1 infection

The HIV-1-mediated decrease in CD1c expression and the subsequent decrease in IFN-γ secretion by CD1c-restricted T cells suggested that HIV-1 downregulates CD1c in an effort to evade the host immune response. To test this, we cultured HIV-1-infected cells with CD1c-restricted T cells for 24 hours and measured viral protein p24 production. In the presence of CD1c-restricted T cells, less viral p24 was produced than by infected cells alone (Figure 6B). Thus, while the HIV-1-mediated decrease in CD1c expression appears to be an immune evasion mechanism used by the virus to decrease the CD1c-restricted T-cell reactivity, these attempts appear to be ineffective for suppressing the anti-viral response mediated by the CD1c-restricted T cells.

### Effect of HIV-1 infection on CD1c/CD1d expression by primary cells and the subsequent CD1c-restricted primary T-cell response

**Figure 7:**
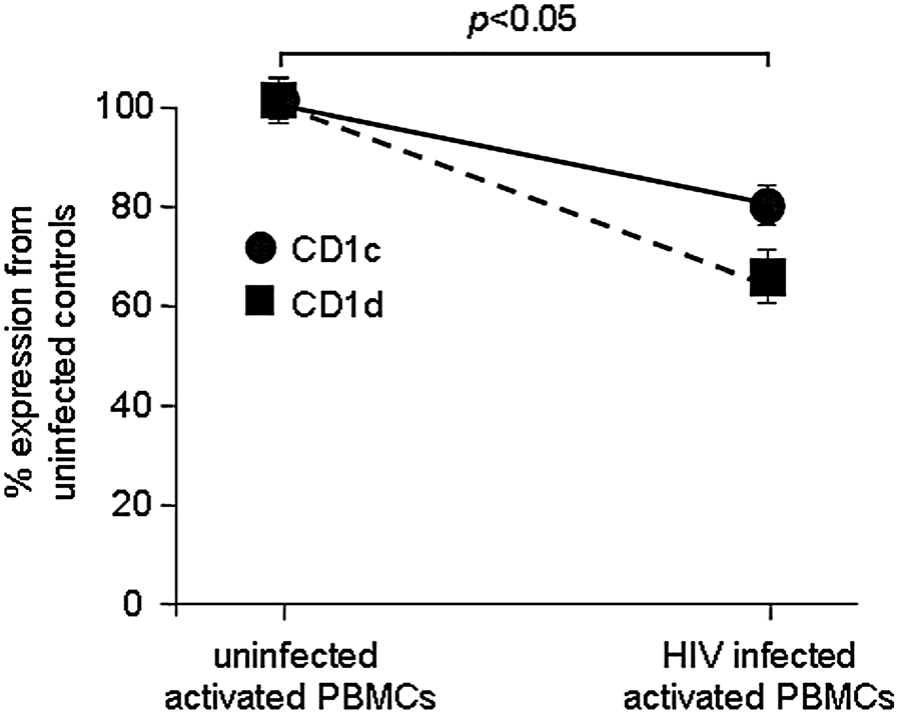
**HIV modulates CD1c/CD1d expression on primary cells.** Peripheral blood mononuclear cells (PBMCs) from healthy donors were isolated, activated with phytohaemagglutinin (PHA), and mock or HIV-1 IIIb infected for 10 days. The expression levels of CD1c and CD1d were measured by FACS. The levels of CD1c/CD1d expression were normalized using uninfected, treated controls and plotted as % modulation from controls. Data represent results from one of three independent experiments.

For the previous studies, we used Jurkat cells and CD1c-restricted T cells that were stimulated for several weeks prior to use. Both cell lines are not ideal as a physiological model. Thus, we assessed CD1c and CD1d expression and endogenous lipid reactivity in primary cells. First, peripheral blood mononuclear cells (PBMCs) isolated from healthy donors were stimulated with 0.5 μg/ml phytohaemagglutinin (PHA) for several days and infected with HIV-1 IIIb, as described in the methods section. The levels of CD1c and CD1d expression were measured 10 days later by FACS. We found that, similar to Jurkat cells, there was a decrease in CD1c and CD1d expression on the HIV-infected cells compared to the uninfected controls. However, unlike the Jurkat cells, this decrease was not significant (Figure 7).

**Figure 8:**
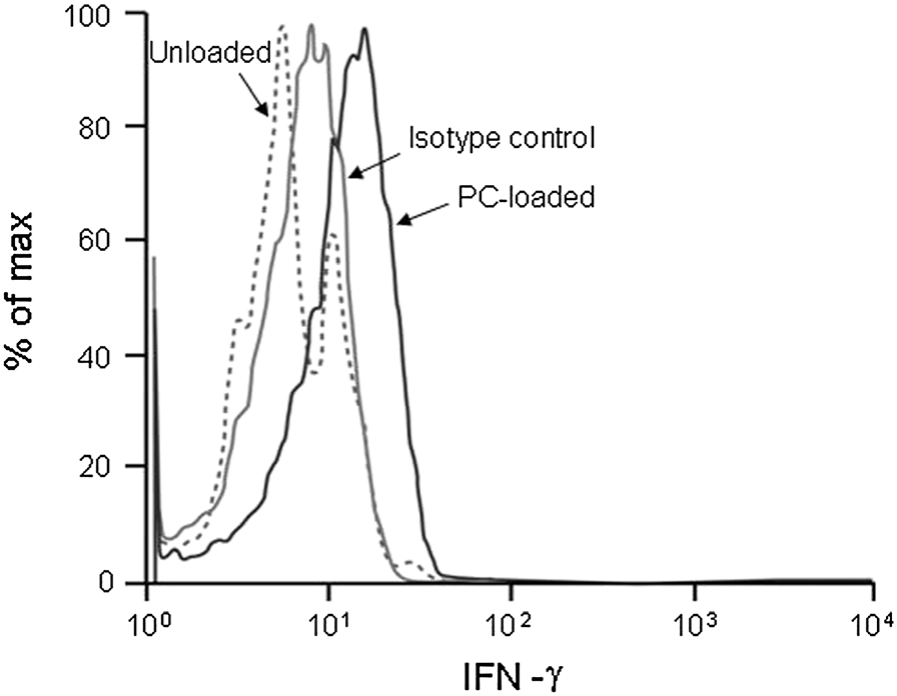
**Endogenous lipid reactive T cells circulate at low levels in the periphery.** Naïve T cells were stimulated with PC-loaded HeLa-CD1c cells overnight. After gating the CD8^+^ population, intracellular IFN-γ was assessed by FACS. A solid black line, a solid grey line, and a dashed line indicate PC-loaded, isotype control, and unloaded, respectively. Data represent results from one of three independent experiments.

We next sought to determine whether endogenous lipid-reactive, CD1c-restricted T cells are present in the peripheral blood. When we stimulated naïve T cells with PC-loaded HeLa-CD1c cells, we found that 1.7% of these naïve cells produced high levels of intracellular IFN-γ (Figure 8). This suggests that endogenous lipid-reactive T cells do circulate at low levels in the periphery and therefore may contribute to anti-HIV-1 immunity during natural infection.

## Discussion

One limitation of anti-HIV immunity is the inability of the MHC-restricted immune response to keep up with the antigenic variability of the virus. The CD1s are MHC-like molecules that present lipid antigens to the immune system. Given the conserved nature of lipids, CD1 presentation of lipids may serve as a less variable antigen presentation model. This could help initiate the immune response before a peptide-restricted response occurs and/or take part in enhancing the adaptive immune response against infection.

In this study, we investigated the role of endogenous lipid presentation during HIV infection. HIV relies heavily on cholesterol for viral production, and in the absence of cholesterol HIV production is impaired [28]. Data suggest that HIV induces the upregulation of several enzymes involved in the cholesterol biosynthesis pathway [29,30] and the synthesis of cholesterol *de novo* within the infected cell [29]. We first assessed whether increased cholesterol synthesis led to an increase in the levels of CD1c and CD1d expression on the cell surface. When Jurkat cells were cultured in the presence of mevalonate, a cholesterol precursor known to increase the production of newly synthesized cholesterol [31], we found that the levels of CD1c and CD1d expression were not increased, but instead maintained, suggesting that cholesterol production may not play a role in upregulating CD1c/CD1d expression. Rather, it appears that increased lipid production helps to maintain CD1c/CD1d expression on the cell surface. This hypothesis was supported by our finding that treatment of HIV-infected cells with the cholesterol inhibitor simvastatin further decreased CD1c/CD1d expression, suggesting that HIV’s dual function in CD1c/CD1d downregulation and increased cholesterol production counteract each other resulting in limited downregulation of CD1c and CD1d molecules from the cell surface.

Similar to what has been shown [23-27], we found that like CD1d, CD1c is downregulated during HIV infection. Contrary to some studies [23,24] and in agreement to one recent study [26], we found that CD1d modulation is Nef-independent. Importantly, the length of the infection may be a factor, as the two studies showing conflicting results to ours assessed CD1d expression after 12 hours of infection, but ours and the other study [26] assessed CD1d modulation more than 2 days post-infection. Furthermore, under these experimental conditions, we found that the downregulation of CD1c and CD1d expression was Vpu-dependent, which is similar to one study that showed Vpu-dependent CD1d downregulation [27]. Finally, we were able to confirm that Vpu itself is sufficient to downregulated both CD1c and CD1d by transfection experiments. Nevertheless, further work is needed to fully understand how Vpu, Nef, or other viral proteins play a role in modulating CD1c and CD1d expression during HIV infection.

We found that CD1c modulation affected the stimulation of CD1c-restricted T cells, and this capacity to stimulate CD1c-restricted T cells was further decreased when CD1c expression was more significantly downregulated. This suggested that HIV intentionally downregulated CD1c molecules in an effort to reduce the amount of IFN-γ secreted by CD1c-restricted T cells. However, these attempts were not entirely effective, as the lowered IFN-γ released in the presence of lowered CD1c expression was still able to significantly reduce viral production. This raises the question whether the composition of endogenous lipids being presented drives the CD1c-restricted T-cell response. This issue will need to be clarified and resolved in a future study.

Our findings are the first to describe endogenous lipid presentation and the functions of CD1c-restricted T cells during HIV-1 infection. Recent data have shown that HIV-1 Nef impairs cholesterol efflux from macrophages [32]. Because of the HIV-1-mediated increase in cholesterol production [29,30], even more cholesterol may be sequestered within the cell. Thus, our data showing CD1c downregulation during HIV-1 infection may indicate one of the HIV-1 immune evasion mechanisms. However, despite the HIV-1-mediated decrease in CD1c expression, the attempts made by HIV-1 to decrease CD1c-restricted T-cell activity were ineffective at completely suppressing the antiviral response. Taken together with our finding that the concurrent cholesterol production induced by HIV-1 virus decreased the extent of CD1c modulation, these data may be useful for exploiting the maintained CD1c levels expressed during HIV-1 infection.

## Conclusions

We found that HIV-1 infection induced the downregulation of CD1c and CD1d expression through a Vpu-dependent, Nef-independent mechanism, and the concomitant HIV-1-induced production of host cholesterol decreased the extent of CD1c and CD1d modulation. These two conflicting HIV-1-mediated actions toward CD1c expression appear to minimize the modulation of CD1c expression, thus leading the host to maintain a CD1c-restricted T-cell response against HIV-1.

## Methods

### Antibodies, cells, plasmids and viruses

Anti-CD1c antibody was purchased from Ancell (Bayport, MN) and Biolegend (San Diego, CA). Monoclonal antibodies against CD1d and HLA-A, B, and C were purchased from BD Biosciences (San Diego, CA). The anti-human IFN-γ antibody was purchased from Abcam (Cambridge, MA). The human Jurkat cell line was obtained from ATCC (Manassas, VA). Cells were cultured in complete RPMI (CRPMI) medium consisting of RPMI 1640 containing 10% fetal calf serum (FCS), penicillin (50 U/ml), streptomycin (50 μg/ml), 2 mM L-glutamine, 1.5 g/L sodium bicarbonate, 4.5 g/L glucose, 10 mM HEPES, and 1.0 mM sodium pyruvate. The cells were incubated in 5% CO_2_ at 37°C. HIV-1 IIIB was obtained from the National Institutes of Health AIDS Research and Reference Reagent Program. A WT plasmid encoding an entire HIV-1 sequence and a PCR3.1 plasmid encoding only HIV-1 Vpu under the CMV promoter were provided by Dr. Paul Bieniasz (Aaron Diamond AIDS Research Center and Rockefeller University). HIV-1 NL4-3 delta Nef (ΔNef), HIV-1 delta Vpu (ΔVpu) and WT viruses were kindly provided by Dr. Derya Unutmaz (New York University School of Medicine), Dr. Paul Bieniasz and Dr. Hiroshi Mori (Aaron Diamond AIDS Research Center), respectively.

### Endogenous lipids and other compounds

PC was purchased from Avanti Polar Lipids, Inc. (Alabaster, AL). Mevalonate and simvastatin were purchased from Sigma Aldrich (St. Louis, MO).

### Transfection and infection of cells

As for the transfection, Jurkat cells were resuspended to 2 x 10^5^ cells/ml in CRPMI medium and placed in a 12-well plate prior to the transfection in a final volume of 1 ml per well. Transfection was performed using plasmid encoding for GFP and the full-length HIV genome (WT) or PCR3.1 vector encoding only Vpu of HIV-1. One μg of DNA was diluted in 200 μl of Optimem serum free media and incubated at room temperature for 15 minutes. After the incubation, 4 μl of Lipofectamine (Invitrogen) was added to the diluted DNA and incubated for 25 more minutes. The DNA-Lipofectamine complex was added to the cells dropwise and incubated for 24 hours at 37°C with 5% CO_2_. After the incubation, cells were harvested and checked for the expression of CD1c and CD1d by a flow cytometric analysis. As for the infection, 2 x 10^6^ Jurkat cells in 10 ml CRPMI were incubated with virus (50TCID_50_) for 2 hours in 10-ml cell culture flasks with gentle mixing every 15–30 minutes. The cells were then incubated for the appropriate number of days at 37°C with 5% CO_2_.

### Assessment of viral load by quantitative RT-PCR

After isolating total RNA from Jurkat cells infected with corresponding HIV-1 viruses using RNAzol reagent (Invitrogen), the RNA was used for RT-PCR reactions. Ten-fold serial dilutions of plasmid containing the fragment of the HIV-1 genome, ranging from 1 to 10^7^ HIV-1 copies per tube, were used to construct the HIV-1 RNA calibration curve. Ten-fold serial dilutions of plasmid containing the fragment of the human GAPDH gene, ranging from 1 to 10^7^ copies per tube, were used to construct the GAPDH calibration curve. The number of RNA copies used in the reaction was then normalized based on the number of GAPDH copies. The quantitative PCR performed with primers and a probe specific for HIV-1 gag were; Forward primer; 5^′^ATC AAG CAG CCA TGC AAA TGT T3^′^(578–599); Reverse primer; 5^′^CTG AAG GGT ACT AGT AGT TCC TGC TAT ATC3^′^(722–752), and Probe; 5^′^FAM-ACC ATC AAT GAG GAA GCT GCA GAA TGG GA-TAMRA3^′^(607–636). GAPDH quantitative PCR was performed with Forward primer; 5^′^GAA GAT GGT GAT GGG ATT TC3^′^, Reverse primer; 5^′^GAA GGT GAA GGT CGG AGT C3^′^, and Probe; 5^′^VIC-CAA GCT TCC CGT TCT CAG CC-TAMRA3^′^.

### Assessment of CD1c/CD1d expression by a flow cytometric analysis

Jurkat cells were harvested and stained for CD1c/CD1d expression for 45 minutes on ice with APC-Cy7-labeled anti-human CD1c antibody (Biolegend) and with PE-labeled anti-human CD1d antibody (BD Biosciences), according to the manufacturer’s instructions. The cells were then washed twice in FACS buffer containing 1× phosphate buffered saline, 2% fetal bovine serum, and fixed in 1% paraformaldehyde. The Mean Fluorescence Intensity (MFI) of both CD1c and CD1d on the GFP-positive Jurkat cells was evaluated on the transfected cells and compared to that on the untransfected controls. Samples were acquired and analyzed using DIVA software on a LSR II (BD, San Jose, CA, USA).

### Generation of CD1c-restricted T cells

To generate the CD1c-restricted T cells, CD14^+^ cells were isolated from PBMCs, and immature dendritic cells (*i*DCs) were generated from the CD14^+^ cells after a 3-day incubation in the presence of 300 U/ml granulocyte-macrophage colony-stimulating factor and 100 U/ml interleukin (IL)-4. The *i*DCs were treated with mitomycin C (50 μg/ml) for 1 hour followed by four washes with culture media. The iDCs were preloaded with PC for 1 hour followed by the addition of the isolated T cells. Six days later, 10 U/ml IL-2 was added, and 30 U/ml IL-2 was added 3 days later or as needed. The cells were cultured for a total of 15 days, re-stimulated for an additional 7–10 days, tested for functionality, and used in experiments.

### Assessment of the CD1c-restricted T-cell response

HeLa cells were transfected with expression vectors encoding CD1c and CD1d glycoproteins (kindly provided by Dr. Shiratsuchi at Aaron Diamond AIDS Research Center) using Lipofectamine 2000 reagent (Invitrogen, Carlsbad, CA) according to the manufacturer’s instructions. These HeLa-CD1c cells were then loaded with PC (50 μg/ml) and used to stimulate the T cells overnight. The next day, supernatants were collected, and IFN-γ levels were assessed by enzyme-linked immunosorbent assay (ELISA). For the experiments, CD1-restricted T cells (5 × 10^5^) were co-cultured with 5 × 10^5^ Jurkat cells, which were either pre-loaded for 1 hour with the indicated amounts of lipid, unloaded, or infected with HIV. The cells were incubated with or without treatment with an inducer or inhibitor of cholesterol synthesis, as described. After culture for 24 hours, supernatants were collected, and IFN-γ secretion was assessed by ELISA. Where applicable, supernatants were also assessed for p24 antigen production by ELISA according to the manufacturer’s instructions (Beckman Coulter, Brea, CA).

### Measurement of cholesterol production

Two million uninfected or HIV-1-infected cells were lysed with 0.1% Triton X solution and used to assess cholesterol production using the Amplex Red Cholesterol Assay Kit (Molecular Probes, Eugene, OR) according to the manufacturer’s instructions.

### Expression of CD1c/CD1d molecules in activated primary cells

PBMCs were isolated from healthy donors and stimulated with 0.5 μg/ml PHA for 3 days. Two million of these cells were then infected with HIV-1 IIIb, HIV-1 NL4-3, HIV-1 NL4-3 ΔNef, or HIV-1 ΔVpu, as described above, and cultured for 2 or 10 days. The level of CD1c/CD1d expression was then assessed on days 2 or 10 post-infection, as described above.

### Assessment of the frequency of PC-reactive CD1c-restricted T cells among PBMCs

T cells were isolated from PBMCs from healthy donors and stimulated with HeLa-CD1c cells that were preloaded with PC (100 μg/ml). After 1 hour, Brefeldin was added, and the cells were cultured for an additional 16 hours. The next day the cells were gated for the CD8 positive population and stained for intracellular IFN-γ, as assessed by a flow cytometric analysis.

### Statistical analysis

The Student’s *t-*test was used to compare differences among the different experimental conditions tested.

## Abbreviations

DC: Dendritic cell; HIV: Human immunodeficiency virus; IFN: Interferon; *i*NKT: Invariant natural killer T; MHC: Major histocompatibility complex; CD: Cluster of differentiation; AIDS: Acquired immune deficiency syndrome; PC: Phosphatidylcholine; WT: Wild-type; PHA: Phytohaemagglutinin; PBMC: Peripheral blood mononuclear cell; *i*DC: Immature dendritic cell; PC: Phosphatidylcholine.

## Competing interests

The authors declare that they have no competing interests.

## Authors’ contributions

HK, RM and JCG carried out the experiments and analyzed the data. HK and MT designed the study, analyzed and interpreted the data and drafted the manuscript. All authors read and approved the final manuscript.

## References

[B1] Brigl M Brenner MB CD1: antigen presentation and T cell function Annu Rev Immuno 2004 22 817 890 10.1146/annurev.immunol.22.012703.104608 15032598

[B2] Moody DB Zajonc DM Wilson IA Anatomy of CD1-lipid antigen complexes Nat Rev Immunol 2005 5 5 387 399 10.1038/nri1605 15864273

[B3] Tsuji M Glycolipids and phospholipids as natural CD1d-binding NKT cell ligands Cell Mol Life Sci 2006 63 16 1889 1898 10.1007/s00018-006-6073-z 16794785 PMC11136056

[B4] Brigl M Bry L Kent SC Gumperz JE Brenner MB Mechanism of CD1d-restricted natural killer T cell activation during microbial infection Nat Immunol 2003 4 12 1230 1237 10.1038/ni1002 14578883

[B5] De Libero G Moran AP Gober HJ Rossy E Shamshiev A Chelnokova O Mazorra Z Vendetti S Sacchi A Prendergast MM Bacterial infections promote T cell recognition of self-glycolipids Immunity 2005 22 6 763 772 10.1016/j.immuni.2005.04.013 15963790

[B6] Gumperz JE Roy C Makowska A Lum D Sugita M Podrebarac T Koezuka Y Porcelli SA Cardell S Brenner MB Behar SM Murine CD1d-restricted T cell recognition of cellular lipids Immunity 2000 12 2 211 221 10.1016/S1074-7613(00)80174-0 10714687

[B7] Vincent MS Xiong X Grant EP Peng W Brenner MB CD1a-, b-, and c-restricted TCRs recognize both self and foreign antigens J Immunol 2005 175 10 6344 6351 16272286 10.4049/jimmunol.175.10.6344

[B8] Porcelli S Brenner MB Greenstein JL Balk SP Terhorst C Bleicher PA Recognition of cluster of differentiation 1 antigens by human CD4-CD8-cytolytic T lymphocytes Nature 1989 341 6241 447 450 10.1038/341447a0 2477705

[B9] Bendelac A Lantz O Quimby ME Yewdell JW Bennink JR Brutkiewicz RR CD1 recognition by mouse NK1+ T lymphocytes Science 1995 268 5212 863 865 10.1126/science.7538697 7538697

[B10] Exley M Garcia J Balk SP Porcelli S Requirements for CD1d recognition by human invariant Valpha24+ CD4-CD8- T cells J Exp Med 1997 186 1 109 120 10.1084/jem.186.1.109 9207002 PMC2198960

[B11] Kawashima T Norose Y Watanabe Y Enomoto Y Narazaki H Watari E Tanaka S Takahashi H Yano I Brenner MB Sugita M Cutting edge: major CD8 T cell response to live bacillus Calmette-Guerin is mediated by CD1 molecules J Immunol 2003 170 11 5345 5348 12759406 10.4049/jimmunol.170.11.5345

[B12] Nieuwenhuis EE Matsumoto T Exley M Schleipman RA Glickman J Bailey DT Corazza N Colgan SP Onderdonk AB Blumberg RS CD1d-dependent macrophage-mediated clearance of Pseudomonas aeruginosa from lung Nat Med 2002 8 6 588 593 10.1038/nm0602-588 12042809

[B13] Porcelli S Morita CT Brenner MB CD1b restricts the response of human CD4-8- T lymphocytes to a microbial antigen Nature 1992 360 6404 593 597 10.1038/360593a0 1281285

[B14] Rosat JP Grant EP Beckman EM Dascher CC Sieling PA Frederique D Modlin RL Porcelli SA Furlong ST Brenner MB CD1-restricted microbial lipid antigen-specific recognition found in the CD8+ alpha beta T cell pool J Immunol 1999 162 1 366 371 9886408

[B15] Sieling PA Ochoa MT Jullien D Leslie DS Sabet S Rosat JP Burdick AE Rea TH Brenner MB Porcelli SA Modlin RL Evidence for human CD4+ T cells in the CD1-restricted repertoire: derivation of mycobacteria-reactive T cells from leprosy lesions J Immunol 2000 164 9 4790 4796 10779786 10.4049/jimmunol.164.9.4790

[B16] Shamshiev A Gober HJ Donda A Mazorra Z Mori L De Libero G Presentation of the same glycolipid by different CD1 molecules J Exp Med 2002 195 8 1013 1021 10.1084/jem.20011963 11956292 PMC2193693

[B17] Vincent MS Leslie DS Gumperz JE Xiong X Grant EP Brenner MB CD1-dependent dendritic cell instruction Nat Immunol 2002 3 12 1163 1168 10.1038/ni851 12415264

[B18] Leslie DS Vincent MS Spada FM Das H Sugita M Morita CT Brenner MB CD1-mediated gamma/delta T cell maturation of dendritic cells J Exp Med 2002 196 12 1575 1584 10.1084/jem.20021515 12486100 PMC2196072

[B19] Carnaud C Lee D Donnars O Park SH Beavis A Koezuka Y Bendelac A Cutting edge: Cross-talk between cells of the innate immune system: NKT cells rapidly activate NK cells J Immunol 1999 163 9 4647 4650 10528160

[B20] Sanchez DJ Gumperz JE Ganem D Regulation of CD1d expression and function by a herpesvirus infection J Clin Invest 2005 115 5 1369 1378 15864354 10.1172/JCI24041 PMC1087176

[B21] Yuan W Dasgupta A Cresswell P Herpes simplex virus evades natural killer T cell recognition by suppressing CD1d recycling Nat Immunol 2006 7 8 835 842 10.1038/ni1364 16845396

[B22] Grubor-Bauk B Simmons A Mayrhofer G Speck PG Impaired clearance of herpes simplex virus type 1 from mice lacking CD1d or NKT cells expressing the semivariant V alpha 14-J alpha 281 TCR J Immunol 2003 170 3 1430 1434 12538704 10.4049/jimmunol.170.3.1430

[B23] Chen N McCarthy C Drakesmith H Li D Cerundolo V McMichael AJ Screaton GR Xu XN HIV-1 down-regulates the expression of CD1d via Nef Eur J Immunol 2006 36 2 278 286 10.1002/eji.200535487 16385629

[B24] Cho S Knox KS Kohli LM He JJ Exley MA Wilson SB Brutkiewicz RR Impaired cell surface expression of human CD1d by the formation of an HIV-1 Nef/CD1d complex Virology 2005 337 2 242 252 10.1016/j.virol.2005.04.020 15916790

[B25] Hage CA Kohli LL Cho S Brutkiewicz RR Twigg HL 3rd Knox KS Human immunodeficiency virus gp120 downregulates CD1d cell surface expression Immunol Lett 2005 98 1 131 135 10.1016/j.imlet.2004.10.025 15790518

[B26] Leonard JA Filzen T Carter CC Schaefer M Collins KL HIV-1 Nef disrupts intracellular trafficking of major histocompatibility complex class I, CD4, CD8, and CD28 by distinct pathways that share common elements J Virol 2011 85 14 6867 6881 10.1128/JVI.00229-11 21543478 PMC3126561

[B27] Moll M Andersson SK Smed-Sörensen A Sandberg JK Inhibition of lipid antigen presentation in dendritic cells by HIV-1 Vpu interference with CD1d recycling from endosomal compartments Blood 2010 116 11 1876 1884 10.1182/blood-2009-09-243667 20530791 PMC3173986

[B28] Ono A Freed EO Plasma membrane rafts play a critical role in HIV-1 assembly and release Proc Natl Acad Sci U S A 2001 98 24 13925 13930 10.1073/pnas.241320298 11717449 PMC61143

[B29] van 't Wout AB Swain JV Schindler M Rao U Pathmajeyan MS Mullins JI Kirchhoff F Nef induces multiple genes involved in cholesterol synthesis and uptake in human immunodeficiency virus type 1-infected T cells J Virol 2005 79 15 10053 10058 10.1128/JVI.79.15.10053-10058.2005 16014965 PMC1181597

[B30] Zheng YH Plemenitas A Fielding CJ Peterlin BM Nef increases the synthesis of and transports cholesterol to lipid rafts and HIV-1 progeny virions Proc Natl Acad Sci U S A 2003 100 14 8460 8465 10.1073/pnas.1437453100 12824470 PMC166251

[B31] Nilsson A Increased cholesterol-ester formation during forced cholesterol synthesis in rat hepatocytes Eur J Biochem 1975 51 2 337 342 10.1111/j.1432-1033.1975.tb03933.x 1097241

[B32] Mujawar Z Rose H Morrow MP Pushkarsky T Dubrovsky L Mukhamedova N Fu Y Dart A Orenstein JM Bobryshev YV Bukrinsky M Sviridov D Human immunodeficiency virus impairs reverse cholesterol transport from macrophages PLoS Biol 2006 4 11 e365 10.1371/journal.pbio.0040365 17076584 PMC1629034

